# Evaluating plant-available P methods and quality characterization of biochar derived from manure and sewage sludge

**DOI:** 10.1007/s10653-026-03157-0

**Published:** 2026-03-27

**Authors:** Merkeb Woldu Bezabeh, Susanne Eich-Greatorex, Åsgeir R. Almås, Tore Krogstad

**Affiliations:** Faculty of Environmental Sciences and Natural Resource Management, NMBU, 1433 Ås, Norway

**Keywords:** Biochar, P availability, Extraction methods, Fertilizers regulation

## Abstract

Phosphorus(P) is essential yet limited, and Norway, like the rest of Europe, remains dependent on imported phosphate rock. Biochar from pyrolysis organic residues offers a potential alternative P source, although quantifying plant-available P is complicated by diverse P speciation. In this study, biochar produced at 400 °C from raw manure, manure digestate, and biologically treated sewage sludge digestate was evaluated using multiple P extraction methods (Olsen, Mehlich-3, ammonium lactate, citric acid, water, oxalate, dithionite, nitric acid). Pot experiments with wheat, spinach, and faba bean were conducted to assess the relationship between extracted P and plant uptake. Results showed that manure-derived biochars had higher agronomic value than sewage sludge biochar, which was limited by Fe/Al-bound P and heavy-metal contamination. Concentrated nitric acid digestion best predicted wheat and faba bean P uptake, while the more common extractants for agronomic evaluation (Mehlich-3, Ammonium Lactate, Olsen, DI-water extracts correlated most strongly with spinach uptake. Digested manure biochar met regulatory requirements for trace metals, but raw manure and sewage sludge biochar exceeded Ni and Zn thresholds, respectively. These findings highlight the need for a tiered approach to biochar P testing and for regulatory frameworks that balance environmental safety with the potential for nutrient recycling.

**Supplementary Information:**

The online version contains supplementary material available at 10.1007/s10653-026-03157-0.

## Introduction

Phosphorus (P) is an essential nutrient for plant growth, playing a key role in energy transfer, photosynthesis, and root development (Lambers, 2021; Poirier et al., 2022; Sharma et al., 2018). However, P availability in soil is often low due to its strong sorption to soil particles, making it less accessible to plants (Frossard et al., 2000; Zhu et al., 2018).

In agriculture, P fertilizers are commonly used; however, excessive application can lead to environmental issues, such as water pollution and eutrophication (Ahmad et al., 2023; Bindraban et al., 2020; Zhang et al., 2018). Additionally, Europe, including Norway, relies heavily on imported phosphate rock, which is a non-renewable resource (Mew et al., 2019; Soesoo & Kirsimäe, 2020). Researchers are exploring alternative P sources, such as biochar, to address these challenges.

P is a critical concern in Europe, including Norway, due to its reliance on imported phosphate rock, a finite and geopolitically sensitive resource (Van Dijk et al., 2016). The European Union has classified P as a critical raw material, emphasizing the need for sustainable P management and recycling strategies (Hool et al., 2024). Unlike countries with sizable natural P reserves, Europe is vulnerable to phosphorus supply-chain disruptions, increasing the urgency to find and evaluate alternative and secondary P sources, including biochar derived from organic residues.

In Norway, P runoff is a significant environmental concern due to intensive agriculture and high rainfall, which contribute to eutrophication in lakes, rivers, and coastal waters (Bechmann et al., 2021; Bechmanna et al., 2005; Ulén et al., 2007). Excess P from fertilizers can trigger algal blooms, which degrade water quality and disrupt aquatic ecosystems. The Norwegian government has strict environmental policies promoting nutrient recycling to reduce P losses and enhance circular economy practices (Müller et al., 2023).

To address these challenges, there is a need to explore alternative P sources, such as biochar. Biochar is a carbon-rich material produced by heating organic matter without oxygen (pyrolysis) (Amalina et al., 2022). It has been widely studied for its benefits in improving soil quality, e.g., by enhancing water retention, storage of soil C, and acting as a slow-release nutrient source (Almutari, 2023; Rafique et al., 2025; Rashid et al., 2021; Voruganti, 2023). Depending on the feedstock, P contents in biochar can vary widely. Thus, biochar with high P content may be a potential alternative to mineral fertilizer P (Farrar et al., 2018; Mashamaite et al., 2024).

Biochar offers a sustainable solution by enhancing soil P retention, reducing leaching, and minimizing pollution risks (Ghodszad et al., 2021; Xu et al., 2018; Yang et al., 2020). Additionally, its potential role in carbon sequestration aligns with Norwegian and European climate policies (Lehmann & Joseph, 2024). By integrating biochar into P management strategies, Norway can enhance soil fertility, reduce reliance on mineral fertilisers, and contribute to environmental protection.

Assessing P availability in biochar is crucial in determining its fertilization effect. However, accurately measuring the plant-available P in biochar is challenging due to its complex chemical composition, which includes a continuum of P species ranging from readily soluble forms (e.g., orthophosphates and weakly bound calcium (Ca)–P or magnesium(Mg)–P) to more recalcitrant mineral phases (e.g., hydroxyapatite, whitlockite, and iron/aluminum (Fe/Al)–P compounds (Li et al., 2018). The distribution and solubility of these species depend strongly on factors such as feedstock type, pyrolysis temperature, and post-production modifications (Glaser & Lehr, 2019; Musa et al., 2024; Sun et al., 2018; Tesfaye et al., 2021; Yang & Lu, 2021).

As P in biochar exists in various chemical forms, different extraction methods are employed to assess its content and bioavailability. Each method targets specific P fractions, offering insights into how much P is readily available to plants and how much is locked in stable forms. The choice of extraction method significantly influences the measured P content and its interpretation for agricultural purposes. For example, Water extraction recovers the most soluble and immediately plant-available P, which usually represents only a small portion of total P in biochar due to reduced solubility after pyrolysis (Xu et al., 2016).

The Ammonium Lactate (AL) method (Egnér et al., 1960) uses an acidic solution (pH 3.75) to extract labile and exchangeable P, closely related to short-term plant availability (Amery et al., 2021; Blombäck et al., 2021; Braun et al., 2019; Teklić et al., 2009; Vona et al., 2022). AL may dissolve some Fe/Al oxides and Ca phosphates, leading to overestimation of P in calcareous soils (Braun et al., 2019), but remains the standard agronomic test in much of Europe, including Norway (Kristoffersen et al., 2020). The Olsen method uses 0.5 M NaHCO_3_ at pH 8.5 to target labile inorganic P, especially in neutral to alkaline soils (FAO, 2021; Iatrou et al., 2014). Mehlich-3 (M3), a multi-nutrient extractant containing acids, ammonium fluoride, and EDTA, mobilizes P associated with Al, Fe, and Ca compounds. Its efficiency varies with soil pH and mineralogy, but it reliably estimates labile to moderately available P across diverse soils (Fukuda et al., 2017; Iatrou et al., 2014; Penn et al., 2018; Zeitoun et al., 2022). Citric acid extraction simulates root-exuded organic acids, mobilizing P by ligand exchange and metal chelation. (Patgiri & Sanjay-Swami, 2024; Teklić et al., 2009). Oxalate extraction dissolves amorphous Fe/Al oxides, potentially mobilizing P (Florea et al., 2024; Guo & Yost, 1999; McKeague & Day, 1966; Yuan & Lavkulich, 1994). The CBD (citrate bicarbonate dithionite) method further reduces Fe oxides, releasing P bound to both amorphous and crystalline phases (Akinbola et al., 2013; McKeague & Day, 1966; Oyebiyi, 2024).

In addition, in Norway, the agricultural use of biochar and other organic fertilizers are regulated under the Forklift om gjødselvarer mv. Av organisk opphav (hereafter referred to as LMD (Norwegian fertilizer regulation) 2025). This framework classifies fertilizer products based on dry matter content and total phosphorus (P) concentration, while heavy-metal concentrations are regulated independently through mandatory threshold limits that determine legal compliance. Consequently, material may meet nutrient-based classification criteria but still be ineligible for agricultural use if metal limits are exceeded.

Therefore, this study aims to: 1. evaluate the efficiency of different P extraction methods in predicting plant-available P in biochar derived from manure and sewage sludge; 2. compare the extractable P values across methods and determine the variability caused by feedstock type; 3. assess the relationship between extracted P and plant P uptake (in crops such as wheat, spinach, or faba beans). 4. Characterise and evaluate the quality of biochar based on the new fertiliser regulation LMD, (2025).

We hypothesized that: (H1) the amount of P extracted will vary significantly between methods depending on the biochar feedstock (manure vs. sewage sludge), (H2) P extracted from biochar by various methods shows different relationships with plant P uptake across different crops (spinach, wheat, and faba bean), (H3) biochar quality, evaluated using LMD-2025 metrics (P%, heavy-metal classes, and P: metal ratios), differs among biochars.

To test these hypotheses, biochar produced from different feedstocks has been characterised and incorporated into soil in a pot experiment to investigate the biogeochemical behaviour of biochar-derived P and to figure out how effectively various extraction methods (Table 1) reflect plant P uptake.

**Table 1:** Overview of the studied biochar phosphorus (P) extraction methods, summarizing the composition and pH of the extracting solution, the biochar-to-solution ratio, the extraction time and the process of measurement used

Method	Method Extracting solution	Solution pH	Soil-to-solution ratio	Extraction (digestion) time	Method of measurement
Ammonium lactate (AL)		3.75	1:20	1:30 h	ICP
Citric acid		2.25	1:20	2 h	ICP
Dithionite (citrate-bicarbonate-dithionite (CBD)	0.2 mol/L NaHCO_3_, 0.12 mol/L Na_2_S_2_O_4_,0.24 mol/L C_6_H_5_Na_3_O_7_	8.5	1:50	16 h	ICP
Mehlich 3 (M3)	0.015 mol/L NH_4_F, 0.013 mol/L HNO_3_,0.001 mol/L EDTA,0.3 mol/L CH_3_COOH	2.5	1:10	5 min	ICP
Olsen	0.5 mol/L NaHCO_3_	8.5	1:20	30 min	ICP
Oxalate	0.11 mol/L(COONH_4_)_2_ 0.08 mol/L (COOH)_2_,	3	1:50	4 h	ICP
Water extraction	distilled H_2_O	unbuffered	1:20	2 h	ICP
Nitric acid digestion	5 ml Concentrated HNO_3_,1 ml HCl		0.2–.3: 50	2.30 h in 250 °C	ICP
Ashing and H_2_SO_4_ digestion	5 ml of 6 M H_2_SO_4_		1:250	burned at 550 °C in a furnace for 1 h	spectrophotometrically at 880 nm

## Material and methods

### Soil and biochar analysis

The particle size distribution (i.e., percentage of sand, silt, and clay in the soil, Table 3) was determined using the pipette method (Elonen, 1971). Soil and biochar pH were measured using a calibrated pH electrode in 1:2.5 soil-to-water and 1:5 biochar-to-water suspensions, respectively. Dry matter (DM) of biochar was determined by drying samples at 105 °C for 24 h to remove moisture and allow compositional data to be expressed on a dry-weight basis. Loss on ignition (LOI) was quantified by heating the dried biochar at 550 °C for 3 h to estimate the volatile solids fraction, reflecting the remaining organic carbon content and the degree of carbonization achieved during pyrolysis. Total carbon (C) and nitrogen (N) in soil were determined by dry combustion (Nelson & Sommers, 1982) at 1050 °C using a Leco Truespec 628 analyzer (St. Joseph, Michigan, USA), while biochar C and N were analyzed using a Leco CHN-1000 analyzer (St. Joseph, Michigan, USA); both followed the Dumas combustion method (Bremner & Mulvaney, 1982). For total elemental analysis, soil, and biochar samples (0.2–0.3 g) were digested in concentrated ultrapure HNO_3_ at 260 °C for 2.5 h in an UltraClave microwave digestion system (Milestone). The digests were diluted to 50 mL with deionized water before analysis. Major elements, including Potassium (K), Mg, Ca, Fe, sulfur (S), Al, and Zn, were measured using Inductively Coupled Plasma Optical Emission Spectroscopy (ICP-OES) (Agilent 5100 SVDV). In contrast, trace elements such as boron (B), molybdenum (Mo), arsenic (As), cadmium (Cd), cobalt (Co), chromium (Cr), copper (Cu), nickel (Ni), lead (Pb), and mercury (Hg) were quantified using Inductively Coupled Mass Spectrometry (ICP-MS) (Agilent 8800 Triple Q) equipped with a collision reaction cell. Plant-available nutrients were analyzed in soil only. Plant-available P, K, Ca and Mg were extracted with ammonium lactate (0.1 M NH_4_ lactate and 0.4 M CH_3_COOH, pH 3.75) (Egnér et al., 1960) and measured by ICP-OES (PerkinElmer Optima 5300 DV). Sulphate-S was extracted with 0.016 M KH_2_PO_4_ and quantified using ICP-OES. Soil ammonium (NH_4_^+^) and nitrate (NO_3_^−^) were extracted using 2 M KCl and analyzed by flow injection analysis (FIA Star 5000) following ISO 13395:1996 and EN ISO 11732:1997. Total P in soil was obtained from the total digestion described above and measured by ICP-MS. In contrast, total P in biochar was determined using the ashing and sulphuric acid dissolution method of Møberg and Petersen (1982), followed by color development using ascorbic acid and molybdate reagent, and spectrophotometric detection at 880 nm (Table 1).

### Biochar SEM, XRD and FTIR analysis

Biochar samples were produced through pyrolysis at 400 °C from three feedstocks: biologically treated sewage sludge digestate, raw manure, and digestate manure. For scanning electron microscopy (SEM), air-dried and finely ground biochar powders were mounted on aluminum stubs using double-sided conductive carbon tape and coated with a thin layer of platinum by sputter coating. SEM micrographs were captured at 5000 × magnification using a Zeiss EVO50 Scanning Electron Microscope operated at 10 kV with a working distance of approximately 15 mm. Each image included a 10 µm scale bar for reference. The examination focuses on surface morphology and microstructure, identifying porosity and fibrous structures that indicate potential P retention and release mechanisms.

X-ray diffraction (XRD) patterns were obtained using a Bruker D8 Advance diffractometer equipped with Cu Kα radiation at an accelerating voltage of 40 kV and a current of 40 mA. The diffractograms were recorded in one-dimensional detector mode employing a coupled two-theta/theta scan, with a step size of 0.01° and an integration time of 0.2 s, covering a 2θ range of 5–70°. Phase identification was performed using Bruker Diffraction Evaluation (EVA) software and the Crystallographic Open Database (COD). The analysis concentrated on identifying crystalline P-bearing minerals like calcium, iron, and aluminum phosphates to evaluate how feedstock composition and pyrolysis conditions affected P speciation and potential availability. Quantitative analysis was performed using Diffracplus TOPAS 7 software by the Rietveld structure refinement method.

Fourier-transform infrared spectroscopy (FTIR) was used for structural analysis and characterisation of functional groups on the biochar surfaces. Spectra were collected using a Nicolet iS10 FTIR spectrometer equipped with an ATR accessory and diamond crystal plate. The spectra were obtained at a resolution of 0.4 cm ^−1^ in the range from 500 to 4000 cm^−1^. The FTIR analysis targeted the identification of phosphate-related functional groups (P–O vibrations at 1000–1100 cm^−1^) and complementary surface features such as hydroxyl (O–H), carbonyl (C=O), and carbonate (CO_3_^2−^) bands to distinguish organic and inorganic bonding environments in the biochar.

### Pot experiment design and crop management

A controlled pot experiment was conducted in the growth room facilities at the Norwegian University of Life Sciences (NMBU), Ås, Norway. The test crops included wheat (*Triticum aestivum* L., variety Mirakel), spinach (*Spinacia oleracea* L., variety Vroeg Reuzenblad), and faba bean (*Vicia faba* L., variety Louhi), cultivated in loamy soil (Table 2).The soil used for the pot experiment was collected from the NMBU research farm in Ås, Norway (59°39′49″ N, 10°45′38″ E; 69 m a.s.l.), air-dried and sieved prior to pot filling. A total of 63 pots were prepared, with 21 pots allocated to each of the three crops. Each pot had a soil surface area of 0.0213 m2, a depth of 0.15 m, and a capacity of 3 L, containing approximately 3 kg of air-dried loam soil. The experiment was performed under controlled environmental conditions, with a temperature maintained at 20 °C, a relative humidity of 60–70%, and lighting provided by LED grow lamps with a 16-h photoperiod and an intensity of 8000 lx (148 µmol m^−2^ s^−1^). Pots were arranged in a completely randomized block design (CRBD) with three replicates per treatment to minimize the effect of spatial variability within the growth room. Before sowing, essential nutrients (N, K, Mg, S, Ca, Fe, Mn, Cu, Mo, and B) were supplied according to standard crop requirements, excluding P and zinc (Zn), to specifically assess P availability from biochar and prevent Zn toxicity (Table 2). For faba beans, nitrogen was applied at only 40 kg ha^−1^ as a starter dose since legumes do not require additional nitrogen. Seeds were sown at crop-specific rates: wheat at nine plants per pot, equivalent to 400 seeds per m^2^; spinach with three plants per pot; and faba bean with four plants per pot. Biochar amendments were thoroughly incorporated into the soil before sowing. The treatments were biochar derived from biologically treated sewage sludge digestate (BBTSSD) and biochar from digestate manure (BDM) for wheat; and BBTSSD and biochar from raw manure (BRM) for spinach and faba bean. The maximum biochar mass rate (20 t ha^−1^) was chosen to represent the upper limit for realistic on farm application for organic soil amendments, while P-based rates (100–200 kg P ha^−1^) ensured consistent nutrient inputs across biochars. The source of the material for both BRM and BDM is from the farm at Kalnes Agricultural School in Sarpsborg. Table 3 shows the proportions of the soil used for the pot experiment. For the pot experiment, the soil was classified as loam, comprising 44.4% sand, 37.6% silt, and 18.1% clay. The soil pH was acidic at 4.41; after adding lime, it increased to 6.2, with an electrical conductivity of 160 µS cm^−1^, indicating a non-saline soil. The available nutrient analysis showed moderately high P levels (90.3 mg kg^−1^) (Krogstad et al., 2008), moderate potassium (117 mg kg^−1^), calcium (183.5 mg kg^−1^), magnesium (35.9 mg kg^−1^), and sulphur (61 mg kg^−1^). In contrast, available nitrogen levels were very low (2.43 mg kg^−1^ NO_3_^−^ and 5.67 mg kg^−1^ NH_4_^+^). The soil contained 4.84% organic matter and 2.81% organic carbon, with a total nitrogen content of 0.192%. The total P and potassium contents were 1.27 g kg^−1^ and 8.91 g kg^−1^, respectively. The total calcium and magnesium levels were 3.91 g kg^−1^ and 5.67 g kg^−1^, with a total Sulphur level of 0.277 g kg^−1^. High concentrations of total iron (26.7 g kg^−1^) and aluminum (34.6 g kg^−1^) were also detected.

**Table 2:** Biochar treatments and applied nutrients based on crop nutrient standards (mineral fertilizer)

Treatment	Nitric acid digestion-based P (kg ha^−1^)	N	K	Mg	S	Fe	Mn	Cu	Mo	B
		(kg ha^−1^)								
BBTSSD	100	195	195	20	148	17	10	7.7	0.06	3.2
BBTSSD	200	195	195	20	148	17	10	7.7	0.06	3.2
BBTSSD (20 tons/ha)	306	195	195	20	148	17	10	7.7	0.06	3.2
BRM	100	195	195	20	148	17	10	7.7	0.06	3.2
BRM	200	195	195	20	148	17	10	7.7	0.06	3.2
BRM (20 tons/ha)	214	195	195	20	148	17	10	7.7	0.06	3.2
BDM	100	195	195	20	148	17	10	7.7	0.06	3.2
BDM	200	195	195	20	148	17	10	7.7	0.06	3.2
BDM (20 tons/ha)	520	195	195	20	148	17	10	7.7	0.06	3.2

Where BBTSSD, Biochar from biologically treated sewage sludge-digestate; BRM, biochar from raw manure; BDM, Biochar from digestate manure

**Table 3:** Physico-chemical properties of the experimental pot soil

Parameter	Mean ± *Sd*	Parameter	Mean ± Sd
Texture classification	Loam	Organic matter (%) = TOC (%) * 1.724	4.84 ± ***0.02***
Sand%	44.4 ± ***0.87***	Total C (%)	2.81 ± ***0.012***
Silt%	37.6 ± ***0.70***	Total N (%)	0.192 ± ***0.004***
clay%	18 ± ***1.56***	Total P (g kg^−1^)	1.27 ± ***0.027***
pH	4.41 ± ***0.006***	Total K (g kg^−1^)	8.91 ± ***0.176***
PH after liming	6.12		
EC (µS cm^−1^)	160 ± ***17.1***	Total Mg (g kg^−1^)	5.67 ± ***0.029***
P–AL (mg kg^−1^)	90.3 ± 0.577	Total Ca (g kg^−1^)	3.91 ± ***0.035***
K–AL (mg kg^−1^)	117 ± ***11.5***	Total S (g kg^−1^)	0.277 ± ***0.010***
Mg–AL (mg kg^−1^)	35 ± ***9.24***	Total Fe (g kg^−1^)	26.7 ± ***0.265***
Ca–AL (mg kg^−1^)	183 ± ***5.77***	Total Al (g kg^−1^)	34.6 ± ***0.666***
KCl–NO_3_–N (mg kg^−1^)	2.43 ± ***1.07***		
KCl–NH_4_–N (mg kg^−1^)	5.67 ± ***0.289***		
Sulfur phosphate extr. S (mg kg^−1^)	61 ± ***2.08***		

### P plant uptake

P concentration in plant tissue after harvest was measured by acid digestion with nitric acid and determination of P with ICP-MS. P uptake (mg pot-1) in the test crops was calculated after harvest determined by multiplying the P concentration in plant tissues (mg g-1 DW) by their respective dry matter yields (g pot-1). For wheat, total P uptake was calculated as the sum of straw and grain uptake, with straw uptake obtained by multiplying the straw dry weight by the straw concentration. Grain (seed) uptake was calculated separately from the grain fraction. In faba bean, total P uptake was derived from the combined uptake of straw and seed fractions. In contrast, seed uptake was estimated independently from seed dry weight and seed P concentration, reflecting the substantial allocation of nutrients into legume seeds. For spinach, total P uptake was determined solely from the edible leaf biomass, calculated as leaf dry weight multiplied by P concentration. To express results on a field-equivalent basis, uptake values were converted to kilograms per hectare using the relation: Where the pot surface area was 00213 m^2^, this procedure ensured that both total and seed P uptake were accurately quantified for each crop at harvest time.

1 $$P uptake \left(kg h{a}^{-1}\right)=\frac{P uptake \left(mg po{t}^{-1}\right)}{pot area \left({m}^{2}\right)}X\frac{1}{\left({10}^{6}\right)}X10000$$

### Harvesting

Faba bean and wheat plants were harvested at physiological maturity after 120 days and 118 days, respectively. Spinach plants were harvested at the maximum vegetative stage, 28 days after sowing. The harvested spinach samples were oven-dried at 65 °C for 72 h to a constant weight, and the dry biomass was recorded to determine yield. For faba bean and wheat, grain and straw were first separated, oven-dried at 65 °C for 72 h to a constant weight and then weighed to determine dry biomass and grain yield.

### Statistical analysis

Statistical analyses were conducted in R (R Core Team, 2025). Extractable P was compared across biochar types using a two-way ANOVA (factors: extraction method and biochar type, including their interaction). For each crop, linear regression was used to relate plant P uptake to extractable P by different chemical methods. Only relationships with statistically significant regression coefficients (*P* < 0.05) were considered. Extraction methods were then ranked based on the coefficient of determination (R^2^), with higher R^2^ values indicating a stronger ability to predict plant P uptake.

## Result

### Characterisation of biochars used for the experiment

#### Physicochemical and nutrient composition (macro and micronutrients) of biochar

Table 4 shows that the physicochemical and nutrient content of biochar vary significantly depending on the feedstock. The dry matter content was consistently high (> 98%) in all biochars. In contrast, the digestate manure biochar exhibited the most significant loss on ignition (59.8%), followed by raw manure (40.6%) and sewage sludge (40.3%), indicating that a larger proportion of carbonaceous organic matter is in the manure digestate-derived material, after pyrolysis. The pH was highly alkaline in raw manure biochar (11.0), moderately alkaline in digestate manure biochar (9.67), and the lowest in sewage sludge biochar (8.02). Electrical conductivity was highest in the digestate manure biochar (5.24 mS cm^−1^) than in raw manure biochar (3.25 mS cm^−1^) and biological treated sewage sludge biochar (0.75 mS cm^−1^), indicating that the manure-based biochar contained more soluble salts. The carbon and nitrogen contents also varied: total carbon was highest in the digestate manure biochar (47.8%), followed by raw manure biochar (41.2%) and biologically treated sewage sludge digestate biochar (33.6%), while total nitrogen followed the opposite trend, being highest in biological treated sewage sludge digestate biochar (4.54%), intermediate in the digestate manure biochar (2.60%), and lowest in raw manure biochar (1.32%). P was highest in biologically treated sewage sludge digestate biochar (48 g kg^−1^), followed by digestate manure (30.3 g kg^−1^) and raw manure (16.6 g kg^−1^). Potassium was greatest in raw manure biochar (52.2 g kg^−1^), while calcium and magnesium peaked in digestate manure (43.3 and 19.8 g kg^−1^, respectively). Sulphur content was similar in raw manure and sewage sludge biochar (4.6 g kg^−1^) but lower in digestate manure (2.3 g kg^−1^). Among micronutrients, molybdenum was highest in biologically treated sewage sludge digestate (9.8 mg kg^−1^), boron in digestate manure (49.8 mg kg^−1^), and manganese in sewage sludge (0.79 g kg^−1^). Sewage sludge biochar was also strongly enriched in iron (10.4 g kg^−1^) and aluminum (18.3 g kg^−1^), exceeding levels in both manure biochars. Overall, digestate manure biochar was the most nutrient-dense and well-balanced, including high levels of carbon, calcium, magnesium, boron, and P, as well as high alkalinity and electrical conductivity. Raw manure biochar had the highest potassium and pH levels, but the lowest P and micronutrient content. In contrast, sewage sludge biochar had extremely high levels of P, nitrogen, sulfur, iron, aluminum, and molybdenum, but low levels of potassium, calcium, and magnesium, as well as significantly lower pH and electrical conductivity.

**Table 4:** Physicochemical and nutrient composition (macro- and micro-nutrients) of biochars used in the pot experiment

Parameter	Biologically treated sewage sludge *digestate* Mean ± *Sd*	Raw manure Mean ± Sd	Digestate manure Mean ± Sd
Dry matter (%)	99.1	98.7	98.3
Loss on ignition (%)	40.3	40.6	59.8
pH	8.02 ± ***0.02***	11.0 ± ***0.03***	9.67 ± ***0.01***
EC (MS cm^−1^)	0.754 ± ***0.04***	3.25 ± ***1.06***	5.24 ± ***1.39***
Total C (%)	33.6 ± ***0.29***	41.2 ± ***0.75***	47.8 ± ***2.17***
Total N (%)	4.54 ± ***0.13***	1.32 ± ***0.02***	2.60 ± ***0.16***
Total P (g kg^−1^)	48 ± ***1.73***	16.6 ± ***0.57***	30.3 ± ***1.52***
Total K (g kg^−1^)	7.92 ± ***0.03***	52.2 ± ***0.29***	29.3 ± ***0.76***
Total Mg (g kg^−1^)	9.98 ± ***0.03***	9± ***0.10***	19.8 ± ***0.29***
Total Ca (g kg^−1^)	40.3 ± ***0.29***	35.7 ± ***0.29***	43.3 ± ***4.54***
Total S (g kg^−1^)	4.6 ± ***0.050***	4.48 ± ***0.12***	2.32 ± ***0.029***
Total Mo (mg kg^−1^)	9.82 ± ***0.18***	4.9 ± ***0.05***	7.83 ± ***1.03***
Total B (mg kg^−1^)	25.3 ± ***0.29***	35.7 ± ***0.77***	49.8 ± ***4.48***
Total Mn (g kg^−1^)	0.792 ± ***0.01***	0.458 ± ***0.01***	0.647 ± ***0.06***
Total Fe (g kg^−1^)	10.4 ± ***0.58***	8.58 ± ***0.13***	3.8 ± ***0.18***
Total Al (mg kg^−1^)	18.3 ± ***0.29***	6.18 ± ***0.29***	3.95 ± ***0.61***

#### Heavy metal concentrations in biochar

The concentrations of heavy metals (Table 5) and P (Table 6) in the studied biochar varied depending on the feedstock type. Raw manure biochar (400 °C) had a P level of 1.36% (below the 2% threshold for ratio-based regulation). Its levels of Cd, Pb, Hg, Zn, and Cr were all comfortably within the Class 0 limits (LMD, 2025). However, Ni was found to be 76.8 mg kg^−1^, which is higher than the Class II limit (50 mg kg^−1^) and close to the Class III limit (80 mg kg^−1^).Because of this, this biochar was classified as Class III under the dry-matter classification, meaning it cannot be used in agriculture, even when it contains low levels of other metals. Digestate manure biochar (400° C) exhibited elevated Zn (580–720 mg kg^−1^) and Cu (480–600 mg kg^−1^), exceeding Class I but remaining within Class II limits based on the dry-matter system. Its P content exceeded 2%, subjecting it to the P-to-metal ratio rule in the new regulation. The calculated ratios (Table 6) of P: Zn = 35, P: Cu = 42, and P: Ni > 800 were all substantially above the minimum requirements (P: Zn = 19, P: Cu = 24, P: Ni = 310) (LMD, 2025), indicating that the rules were being followed. Because of this, digestate manure biochar meets the requirements for both systems. It is a Class II fertilizer subject to dry-matter restrictions and fully compliant with the ratio-based regulation. Biologically treated sewage sludge digestate biochar (400 °C) contains the highest levels of trace metals. Cd was a value of 1.8 mg kg^−1^ (Class I). Ni reached 70.5 mg kg^−1^ (beyond the Class II limit and near the Class III maximum of 80 mg kg^−1^). Zn was around 1500 mg kg^−1^ (the Class III limit). Cu (468 mg kg^−1^) and Cr (83.5 mg kg^−1^) were higher than usual but still below the Class II limit.

**Table 5:** Heavy metal concentrations in biochars used for the pot experiment

Parameter	Biochar derived from biologically treated sewage sludge digestate Mean ± SD	Biochar from raw manure Mean ± Sd	Biochar from manure digestate Mean ± Sd
Total Cr (mg kg^−1^)	83.5 ± ***10.3***	5.20 ± ***0.00***	9.62 ± ***1.20***
Total Co (mg kg^−1^)	9.77 ± ***0.03***	3.83 ± ***0.19***	2.43 ± ***0.32***
Total Ni (mg kg^−1^)	70.5 ± ***8.85***	76.7 ± ***6.53***	15.2 ± ***2.02***
Total Cu (mg kg^−1^)	468 ± ***5.77***	65.7 ± ***1.26***	116 ± ***21.2***
Total Zn (mg kg^−1^)	1500 ± ***0.00***	452 ± ***5.77***	515 ± ***75.7***
Total As (mg kg^−1^)	4.45 ± ***0.05***	7.07 ± ***0.08***	0.515 ± ***0.03***
Total Cd (mg kg^−1^)	1.80 ± ***0.000***	0.133 ± ***0.00***	0.230 ± ***0.03***
Total Hg (mg kg^−1^)	0.0183 ± ***0.01***	0	0
Total Pb (mg kg^−1^)	41.8 ± ***0.29***	11.5 ± ***0.5***	2.23 ± ***0.32***

**Table 6:** Comparison of phosphorus concentration and P/Heavy metal ratios in different biochar feedstocks

	Biochar from biologically treated sewage sludge digestate	Biochar from manure	Biochar from manure digestate
Total phosphorus(P) %	4.8	1.36	3.03
Ratio of milligrams of phosphorus to milligrams of heavy metals			
P/Cd	26667	102564	133023
P/Pb	1148	1189	13765
P/Hg	2634858		
P/Ni	686	179	2024
P/Zn	32	30	60
P/Cu	102	208	266
P/Cr	579	193	3187
P/As	10785	1934	59244

Despite its high P content (> 1.5%), the P-to-metal ratio system does not apply to biologically treated sewage digestate biochar; hence, classification is determined by the dry-matter thresholds. Based on Zn and Ni levels, this biochar was classified as Class III, disqualifying it from use as an agricultural fertilizer. Overall, manure- and biologically treated sewage digestate biochars differ notably in terms of regulatory compliance: digestate manure biochars adhere to both dry- matter and ratio- based regulations (Class II/full compliance), whereas raw manure and sewage sludge biochars fall into Class III- due to high Ni levels in the former and excessive Zn and Ni in the latter.

### Mineralogical, functional, and morphological characteristics of different biochar

**Fig. 1:**
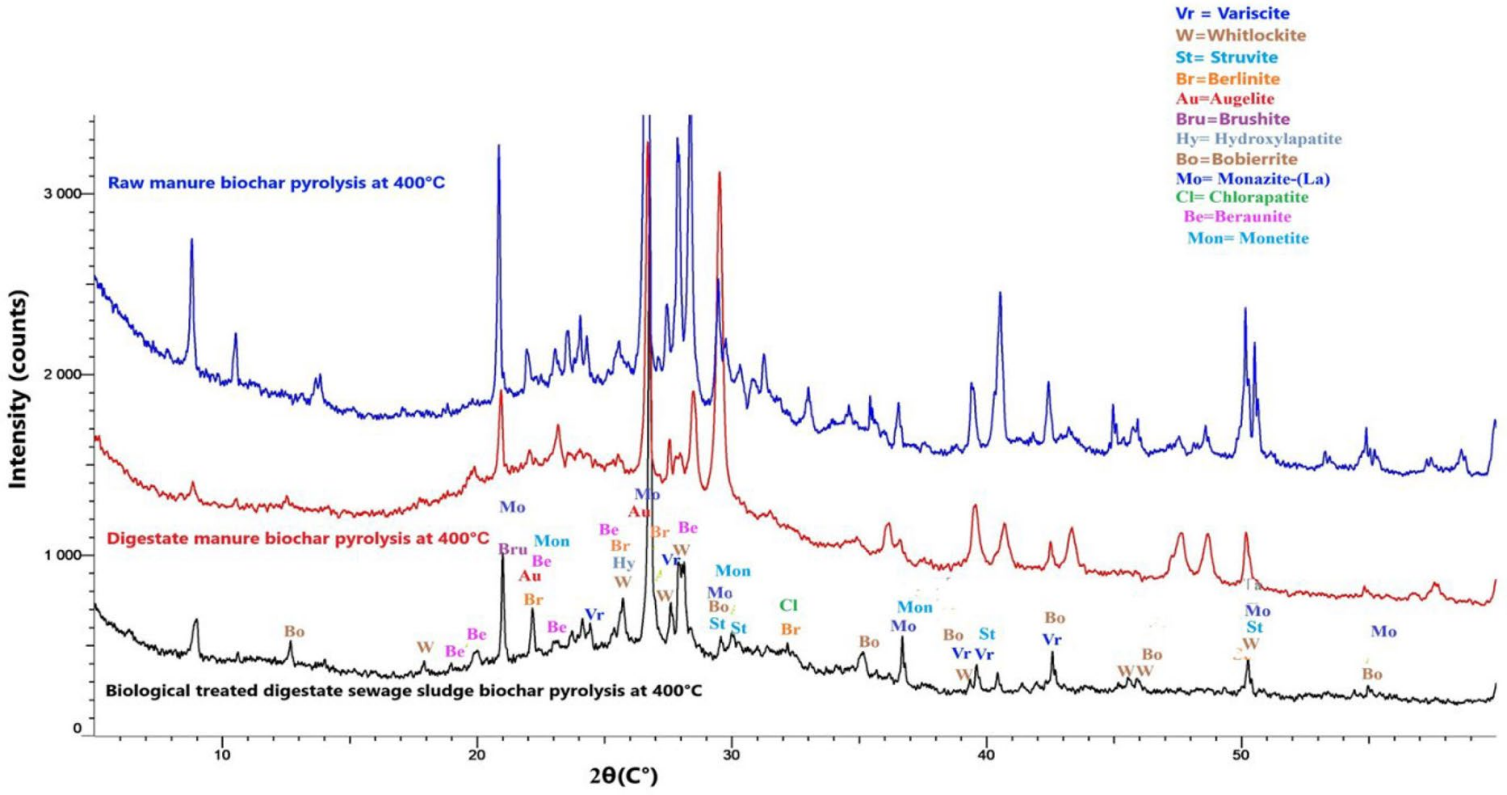
XRD patterns of manure and sewage sludge biochars produced at 400 °C showing different phosphorus-containing minerals

**Fig. 2:**
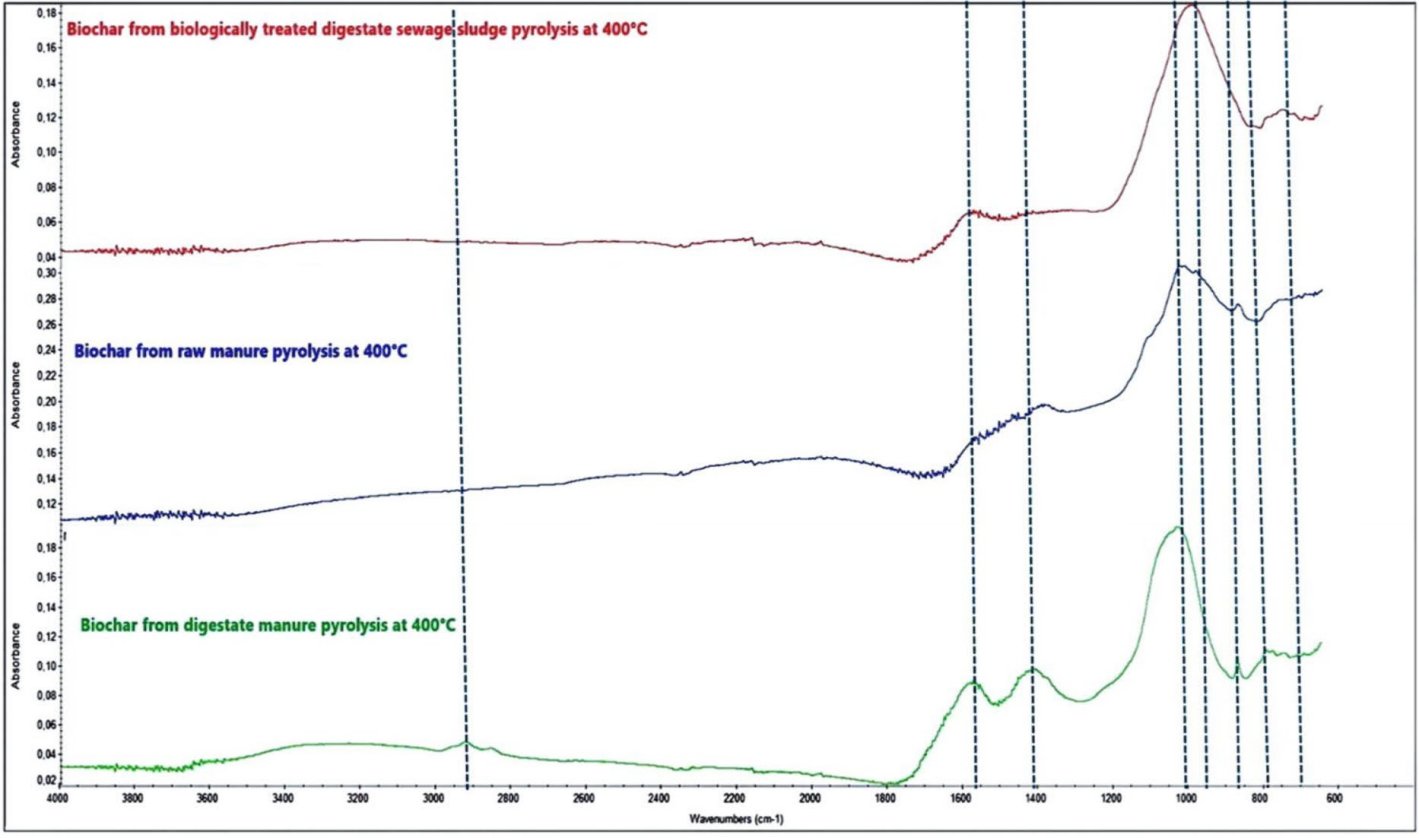
FTIR spectra of biochars from raw manure, digestate manure, and biologically treated sewage sludge produced at 400 °C

**Fig. 3:**
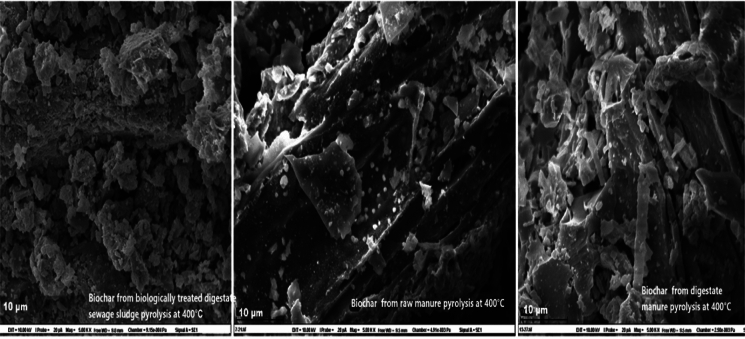
SEM images of biochars produced at 400 °C from biologically treated sewage sludge digestate, raw manure, and digestate manure

X-ray diffraction (XRD) (Fig. 1), quantitative XRD analysis (Table 7), Fourier-transform infrared spectroscopy (FTIR) (Fig. 2), and scanning electron microscopy (SEM) (Fig. 3) analyses discovered apparent differences in mineralogical composition, functional groups, and surface morphology among the three biochars. Raw manure biochar (BRM) was characterized by a high proportion of crystalline Ca-bearing phases, dominated by carbonates (53 wt% of the crystalline fraction) and minor Ca–P minerals (3 wt%), including monetite, brushite, whitlockite, and apatite-type phases. SEM images supported this display of fibrous, porous surfaces with cavities and crystallinity deposits. Digestate manure biochar retained crystalline Ca–P phases but showed a relative enrichment of Ca–P minerals (10 wt% of the crystalline fraction) and a reduced carbonate contribution compared with BRM, indicating mineral transformation during anaerobic digestion. Its morphology was intermediate, with fragmented and flaky structures, partially collapsed pores, and embedded mineral particles. In contrast, the biologically treated digestate sewage sludge biochar was dominated by Al–P (30 wt%) and Fe–P (7 wt%) minerals, including berlinite, variscite, augelite, and iron phosphate phases, with only minor contributions from Ca–P minerals, consistent with its compact and blocky structure, characterized by aggregated mineral-rich surfaces and limited porosity. FTIR spectra further confirmed these trends: all biochar showed strong phosphate-related P–O stretching vibrations at 1000–1100 cm^−1^, while manure-derived biochar retained more organic features, such as O–H stretching (3400 cm^−1^) and carbonyl vibrations (1650 cm^−1^). Digestate manure biochar displayed sharper phosphate bands and additional carbonate absorptions near 1400 cm^−1^, while the sewage sludge biochar exhibited the most intense phosphate absorption, broad O–H stretching, but fewer organic-related bands, reflecting its high ash and mineral content. Together, these results indicate that feedstock type and pretreatment strongly influence the mineral crystallinity, P speciation, functional groups, and surface structures of the biochars.

**Table 7:** Quantitative phase analysis (QPA) of crystalline minerals determined by XRD (Rietveld refinement, TOPAS 7)

Mineral group	Mineral phase	Chemical formula	BBTSSD wt%	BRM wt%	BDM wt%
Al–P minerals	Variscite	AlPO_4_·2H_2_O	0.39	0.37	1.17
	Berlinite	AlPO_4_	29.37	18.56	24.64
	Augelite	Al_2_PO_4_ (OH)_3_	0.55	1.20	1.97
Fe–P minerals	Strengite	FePO_4_·2H_2_O	0.40	0.88	0.64
	Metavivianite	Fe_3_(PO_4_)_2_·xH_2_O	1.00	1.24	0.00
	Beraunite	Fe_6_(PO_4_)_4_O(OH)_4_·6H_2_O	5.74	5.06	1.11
Ca–P minerals	Whitlockite	Ca_9_ (Mg, Fe) (PO_4_)_6_PO_3_OH	0.00	0.11	2.86
	Brushite	Ca HPO_4_·2H_2_O	0.23	0.16	0.22
	Chlorapatite	Ca_5_(PO_4_)_3_Cl	0.36	0.46	0.30
	Hydroxyapatite	Ca_10_(PO_4_)_6_(OH)_2_	0.07	0.01	1.61
	Monetite	CaHPO_4_	1.34	1.69	3.65
Mg–P minerals	Bobierrite	Mg_3_(PO_4_)_2_·8H_2_O	0.18	0.33	0.31
	Struvite	Mg NH_4_PO_4_·6H_2_O	1.54	1.69	0.92
Other phosphates	Monazite	(Ce, La, Nd, Th) PO_4_	0.38	0.97	0.55
Carbonates	Calcite	Ca CO_3_	0.44	53.33	1.69
	Dolomite	Ca Mg (CO_3_)_2_	0.42	0.00	0.39
Silicates & oxides	Muscovite	K Al_2_ (Al Si_3_ O_10_) (OH)_2_	33.29	2.44	5.54
	Albite	Na Al Si_3_ O_8_	22.70	3.44	27.14
	Illite	K_0.65_ Al_4_ (Al_0.65_Si_3.35_O_10_) (OH)_2_	1.12	0.16	5.64
	Amphibole	(Ca, Na)_2_(Mg, Fe, Al)_5_ (Si, Al)_8_O_22_(OH)_2_	1.28	0.40	1.09
	Quartz	Si O_2_	0.34	1.07	1.97
	Hematite	Fe_2_ O_3_	0.25	1.38	0.15
Sulfates & salts	Gypsum	Ca SO_4_·2H_2_O	0.49	0.66	0.99
	Baryte	Ba SO_4_	0.10	0.36	0.12
	Sylvite	KCl	0.03	0.89	3.04
GOF			1.37	1.42	2.47

Where: BBTSSD, biologically treated digestate sewage sludge biochar; BRM, raw manure biochar; BDM, digested manure biochar. Values are expressed as wt% of the crystalline fraction

### Comparing P extraction methods for manure- and sewage sludge-derived biochar

**Fig. 4:**
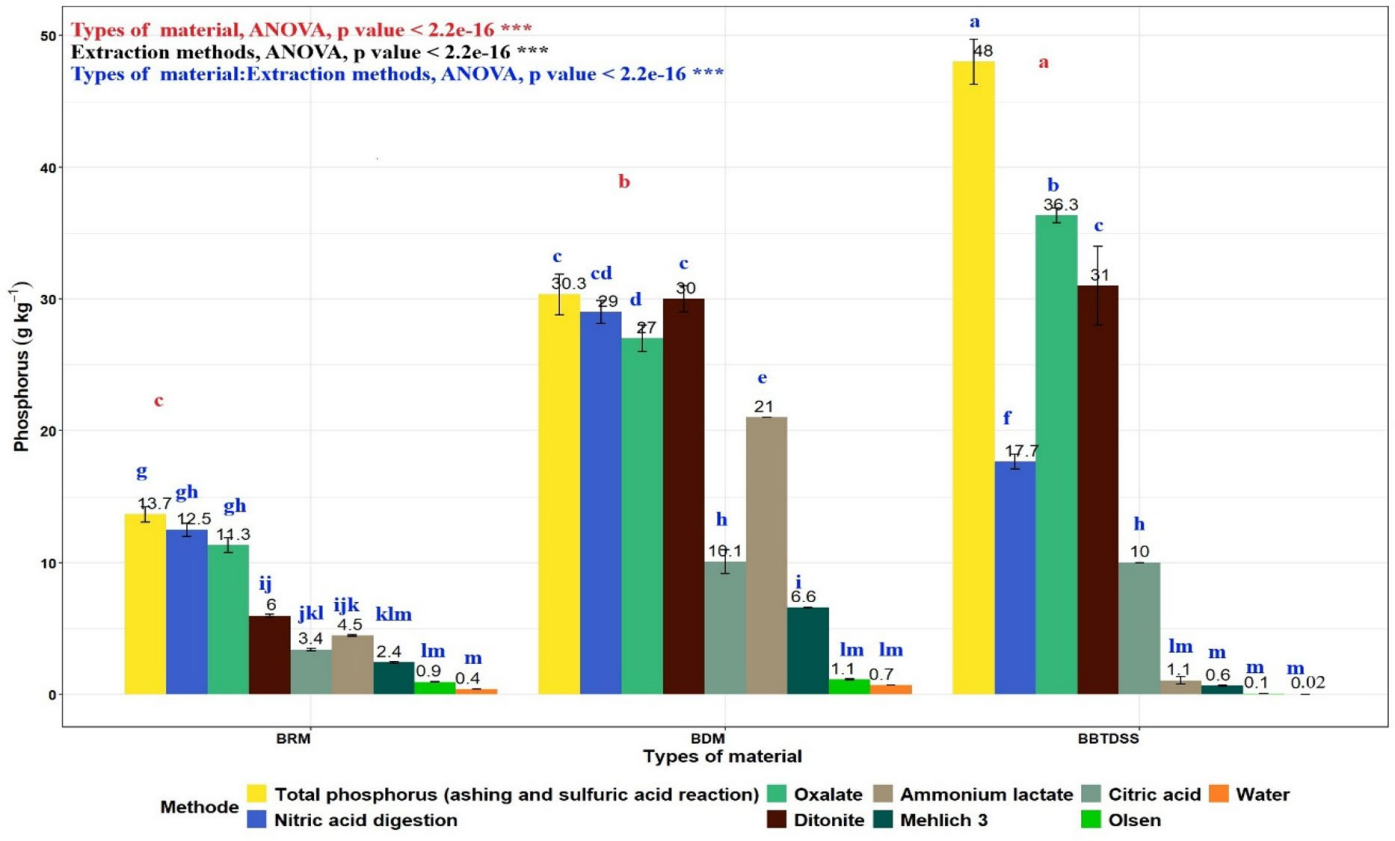
Phosphorus content (g kg^−1^) of biochar from raw manure (BRM), digestate manure(BDM), and biologically treated sewage sludge digestate (BBTSSD) measured by different extraction methods

It was observed that P content in biochar varied depending on extraction method and feedstock type (Fig. 4). Two-way ANOVA revealed significant main effects of feedstock type and extraction methods as well as significant interaction between the two (*P* < 0.001), Indicating that the performance of extraction methods depended strongly on biochar origin.

In raw manure biochar, the highest levels were achieved using the ashing and sulphuric acid reaction (13.7 g kg^−1^) and nitric acid digestion (12.5 g kg^−1^) methods. Mehlich-3 and ammonium lactate extracted substantial amounts (6 g kg^−1^), whereas citric acid, water, and Olsen extracted low concentrations (< 4 g kg^−1^).

In digested manure, biochar was found to contain 30.83 g kg^−1^ using the ashing and sulfuric acid reaction method and 29 g kg^−1^ with nitric acid digestion. Mehlich-3 and ammonium lactate consistently extracted 30 g kg^−1^ and 21 g kg^−1^, respectively. In contrast, biologically treated sewage digestate biochar showed a markedly different extraction pattern. The highest values were obtained with dithionite (31 g kg^−1^) and the ashing and sulphuric acid reaction method (36.33 g kg^−1^). However, very low recoveries were observed with extractants commonly used for agronomic evaluation, such as Olsen, Mehlich-3, and ammonium lactate (< 1.1 g).

### Wheat P uptake in relation to biochar-applied P determined by different extraction methods

**Fig. 5:**
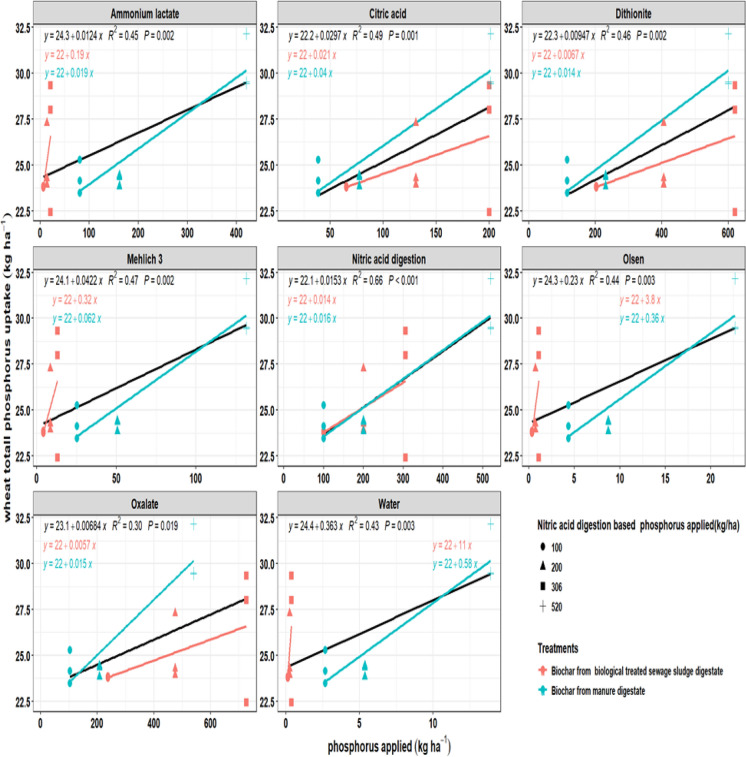
Relationship between applied phosphorus (kg ha^−1^) and wheat total phosphorus uptake (kg ha^−1^) using different extraction methods. Results are shown for biochar from digestate manure (blue) and biologically treated sewage sludge digestate (red). Lines represent regression fits, along with their corresponding R^2^ values and *P*-values

**Fig. 6:**
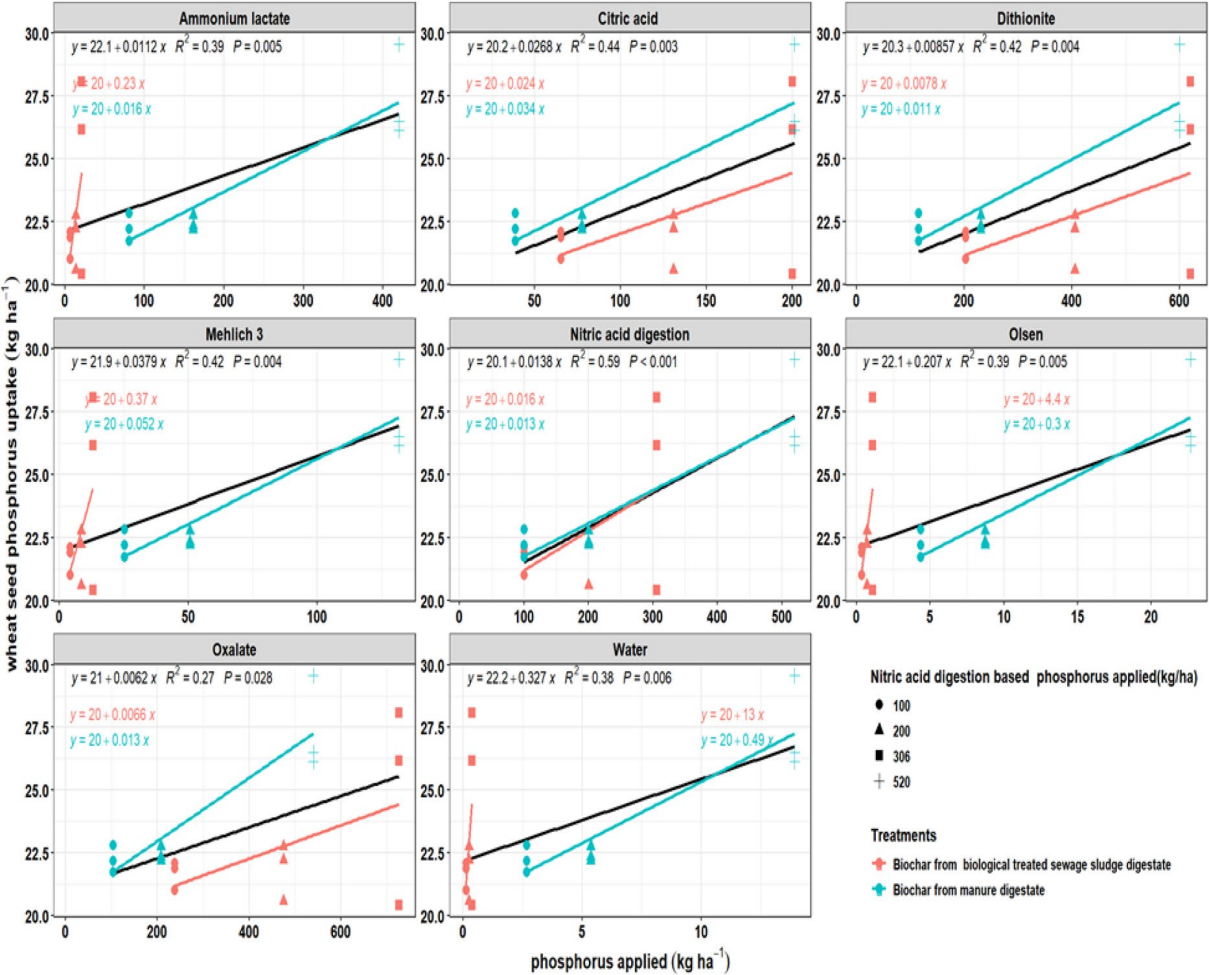
Relationship between applied phosphorus (kg ha^−1^) from different biochars using different extraction methods and wheat seed phosphorus uptake (kg ha^−1^). Results are shown for biochar from digestate manure (blue) and biologically treated sewage sludge digestate (red). Lines represent regression fits, along with their corresponding R^2^ values and *P*-values

The relationship between P (P) application, as estimated by different extraction methods, and wheat P uptake (seed and total) varied depending on the extractant used and the type of feedstock. The black line in the figures represents the average of the red and blue lines, and the statistics R^2^ are based on this line (Figs. 5, 6). In general, P uptake increased with higher application rates; however, the strength of the relationship varied across extraction methods. Nitric acid digestion yielded the strongest correlations with both grain P uptake (R^2^ = 0.59, *P* < 0.001) and total P uptake (R^2^ = 0.66, *P* < 0.001), partly reflecting its role in determining application rates and thus total P input rather than plant-available P alone. Intermediate relationships were observed with citric acid, dithionite, and Mehlich-3 extractions (R^2^ values between 0.42 and 0.49), whereas Olsen, ammonium lactate, and water extractions produced weaker but still significant fits (R^2^ = 0.38–0.45). Oxalate consistently showed the lowest predictive strength (R^2^ = 0.27–0.30), indicating limited capacity to explain plant uptake. When comparing feedstocks, digestate manure biochar exhibited higher regression slopes across all methods except ammonium lactate and the Olsen extraction method, indicating greater release of available P and stronger responsiveness to uptake. In contrast, sewage sludge digestate biochar exhibited lower slopes, indicating restricted P (P) release and a lower contribution to crop P uptake. An exception was observed for ammonium lactate, which overestimated P availability in manure biochar but underestimated it in sewage sludge digestate biochar, as evidenced by higher plant P uptake relative to chemically extractable P in the latter.

### Faba bean P uptake in relation to biochar-applied P determined by different extraction methods

**Fig. 7:**
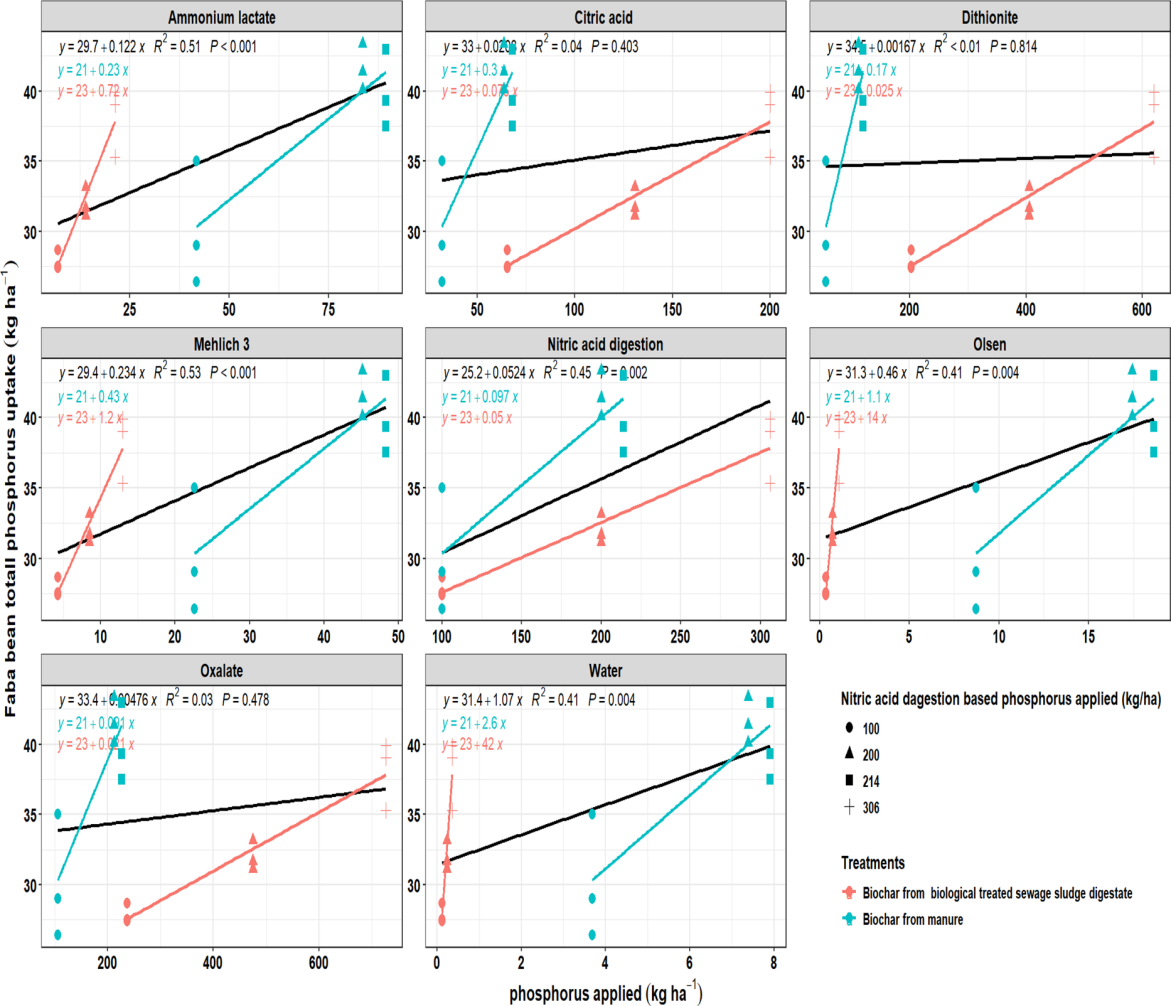
Relationship between applied phosphorus (kg ha^−1^) from different biochars using different extraction methods and faba bean total phosphorus uptake (kg ha^−1^). Results are shown for biochar from digestate manure (blue) and biologically treated sewage sludge digestate (red). Lines represent regression fits, along with their corresponding R^2^ values and *P*-values

**Fig. 8:**
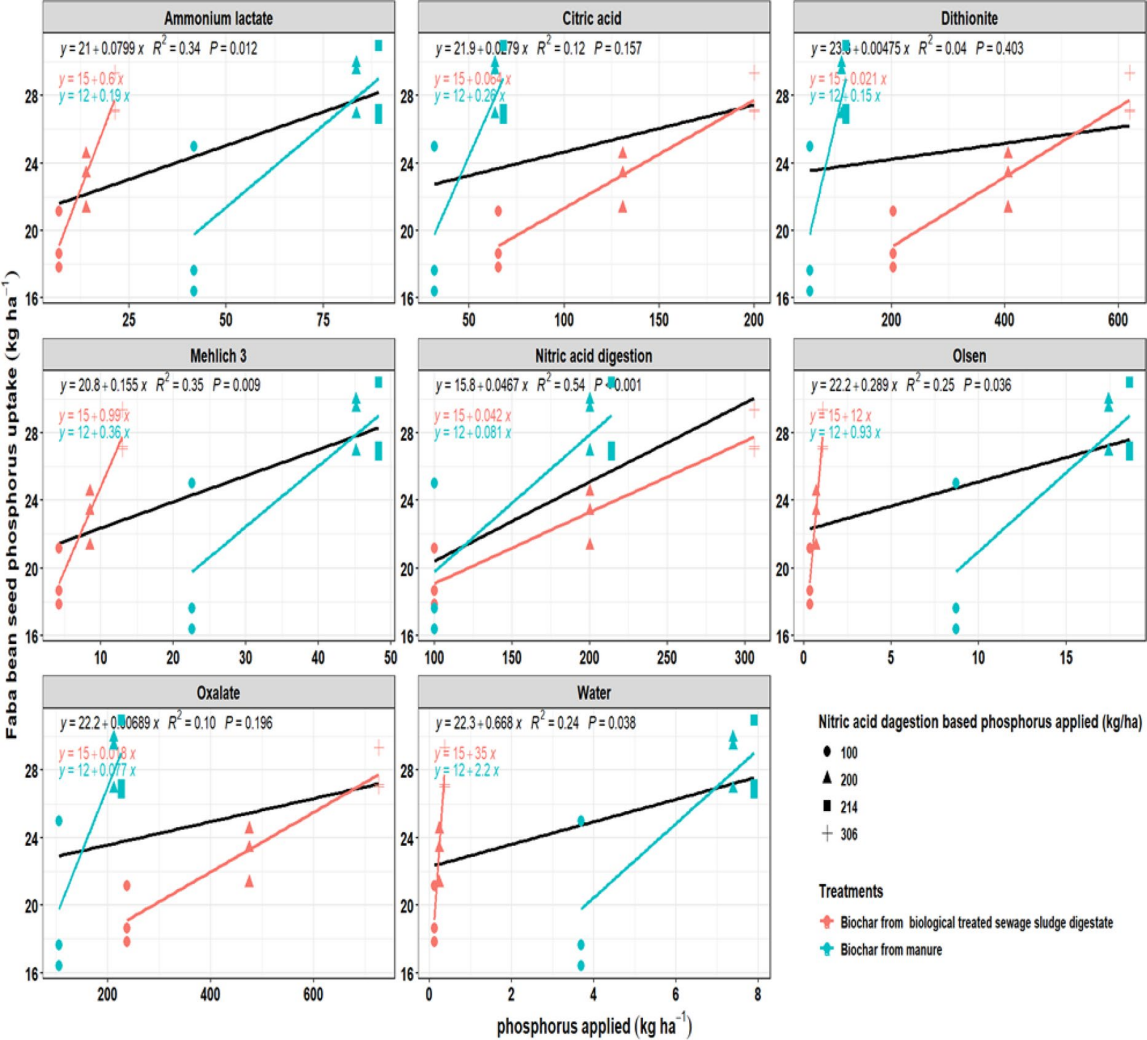
Relationship between applied phosphorus (kg ha^−1^) from different biochars using different extraction methods and faba bean seed phosphorus uptake (kg ha^−1^). Results are shown for biochar from digestate manure (blue) and biologically treated sewage sludge digestate (red). Lines represent regression fits, along with their corresponding R^2^ values and *P*-values

The relationship between P (P) application, as estimated by different extraction methods, and faba bean P uptake (seed and total) varied depending on the extractant used and the type of biochar feedstock (Figs. 7, 8). In general, P uptake increased with higher application rates; however, the strength of the relationship was strongly influenced by the extraction method. For total P uptake, ammonium lactate, Mehl ich 3, nitric acid digestion, Olsen, and water extractions provided significant and moderate-to-strong correlations (R^2^ = 0.41–0.53). In contrast, citric acid, dithionite, and oxalate showed poor predictive strength (R^2^ ≤ 0.04) and non-significant fits. For seed P uptake, nitric acid digestion explained the most significant proportion of variation (R^2^ = 0.54, *P* < 0.001), followed by ammonium lactate, Mehl ich 3, Olsen, and water extractions (R^2^ = 0.24–0.35). Similar to total uptake, extractions using citric acid, dithionite, and oxalate showed weak and non-significant relationships with seed uptake.

When comparing feedstocks, manure-derived biochar consistently exhibited higher regression slopes across most extraction methods, reflecting greater P (P) release and stronger responsiveness to plant uptake. In contrast, sewage sludge digestate biochar resulted in flatter slopes, particularly under extractions with citric acid, dithionite, and oxalate, suggesting that a significant portion of its P remained in less available forms. These findings highlight that the choice of extraction method strongly affects the prediction of P availability from biochar, with Mehl ich 3, and ammonium lactate showing the greatest relevance to faba bean uptake.

### Spinach P uptake in relation to biochar-applied P determined by different extraction methods

**Fig. 9:**
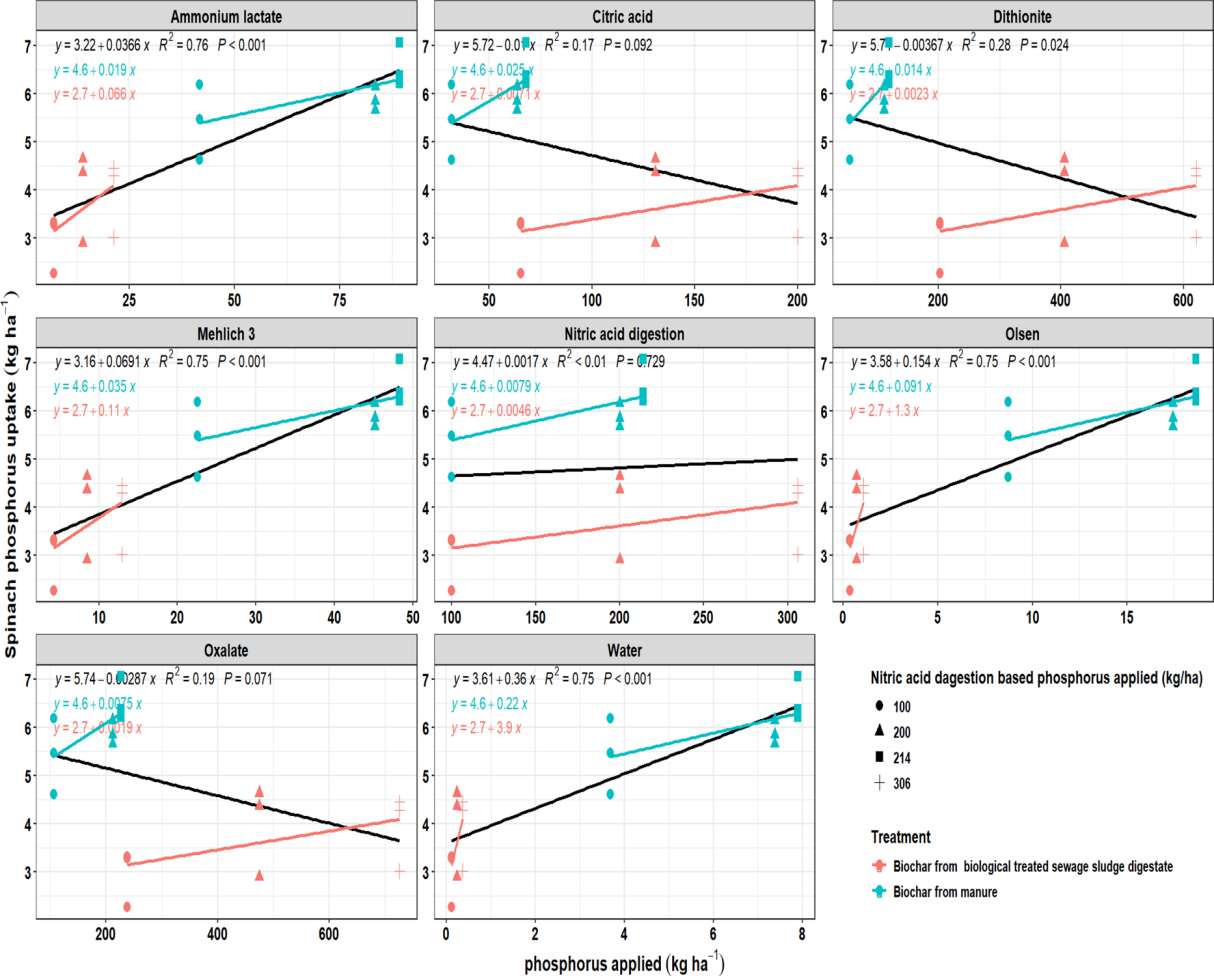
Relationship between applied phosphorus from different biochars using various extraction methods (kg ha^−1^) and spinach phosphorus uptake (kg ha^−1^). Results are shown for biochar from digestate manure (blue) and biologically treated sewage sludge digestate (red). Lines represent regression fits, along with their corresponding R^2^ values and *P*-values

The relationship between P (P) application, estimated by different extraction methods, and spinach P uptake varied across extractants and feedstocks (Fig. 9). Ammonium lactate, Mehlich-3, Olsen, and water extractions showed a strong and significant positive relationship with spinach P uptake (*R*^*2*^ = 0.75–0.76, *P* < 0.001). In contrast, nitric acid digestion revealed no significant relationship (R^2^ < 0.01, *P* = 0.729), suggesting that the P measured by this method did not reflect spinach uptake. Similarly, citric acid, dithionite, and oxalate extractions displayed weak or inconsistent relationships, with low *R*^*2*^ values (0.17–0.28), and in some cases, negative slopes.

Differences were also observed between feedstocks. Spinach P uptake from manure-derived biochar showed clearer linear increases across the stronger extraction methods, while biologically treated sewage sludge biochar resulted in weaker relationships, particularly when assessed by citric acid, dithionite, and oxalate extractions.

## Discussion

### Physicochemical characteristics and nutrient composition of biochar

Biochars effectiveness as a fertiliser is powerfully shaped by its feedstock origin and production conditions. Digestate manure biochar is typically the most nutrient-rich, with high carbon and significant enrichment in P (P), calcium (Ca), magnesium (Mg), and boron (B), making it especially promising for direct fertiliser substitution and for use in acidic soils. However, high electrical conductivity (EC) in these biochars can limit their application in salt-sensitive crops (Meena et al., 2019). Nevertheless, its high P and balanced macronutrient composition are the most promising biochars for direct fertiliser substitution in cropping systems. Biochar made from raw manure is a potential liming material and soil conditioner, offering high alkalinity, nutrient supply, and improved soil properties, with the highest K but much lower P compared to other feedstocks. It is extremely high alkalinity and moderate EC suggest it has strong potential as a liming material and soil conditioner (Bolan et al., 2022). However, its relatively low concentrations of P and micronutrients indicate a more limited role in supplying essential nutrients, and its high K may be more valuable for crops with significant potassium demand. In contrast, biologically treated sewage sludge digestate biochar was strongly differentiated by its lower pH and the lowest EC, reflecting fewer soluble salts despite its high ash content. It contained the highest concentrations of P, N, S, Fe, Al, Mo, and Mn. This enrichment pattern reflects the chemistry of wastewater treatment, which concentrates Fe- and Al-based precipitates that bind P. While the high total P content suggests a strong potential as a P source, its agronomic value is limited because much of the P is immobilised in Fe- and Al-phosphate complexes, reducing solubility and plant availability (Gypser et al., 2021). Additionally, the exceptionally high Fe, Al, and Mo contents raise concerns about potential metal toxicity and long-term soil accumulation. Overall, these results indicate that digestate manure biochar provides the most balanced combination of nutrient supply and soil amendment properties. In contrast, raw manure biochar is more effective as a liming agent with a high K supply. Sewage sludge biochar, despite its high P content, presents challenges due to low pH, limited P availability, and excessive Fe, Al, and Mo, which constrain its use as a fertiliser in agricultural systems.

### Evaluation of biochar fertiliser quality based on dry matter and P to heavy metal ratio limits

The contrasting classification outcomes highlight how feedstock composition and regulatory frameworks jointly determine the fertiliser potential of biochar. Under the traditional dry-matter system, raw manure biochar would be expected to qualify as a high-quality product due to its low levels of Cd, Pb, Hg, Zn, Cu, and Cr. However, the elevated Ni concentration (76.67 mg kg^−1^) pushed this material into Class III, demonstrating how a single metal can determine the regulatory status even when other contaminants are negligible. Because the P concentration of this biochar was only 1.36% of the dry matter, the ratio-based thresholds introduced in the new Norwegian regulation (LMD, 2025) do not apply, leaving Ni as the decisive limiting factor. Digestate manure biochar presented a different case. While Zn (580–720 mg kg^−1^) and Cu (480–600 mg kg^−1^) concentrations exceeded Class I limits, they remained below the Class II thresholds, resulting in a moderate classification under the dry-matter system. More importantly, because its P concentration exceeded 2%, it was assessed under the P-to-metal ratio framework, which serves as a supplementary indicator of nutrient density and permissible application rates. Here, the high P content improved the regulatory standing, as the calculated P: Zn (35), P: Cu (42), and P: Ni (> 800) ratios all exceeded the minimum requirements. In contrast, biologically treated sewage sludge digestate biochar is still problematic. Despite containing abundant P, it is excluded from the ratio-based evaluation because the regulation explicitly exempts products that hold sewage sludge. Consequently, its classification relies on dry-matter thresholds, where Zn (1500 mg kg^−1^) and Ni (70.5 mg kg^−1^) place it in Class III. This outcome reflects the higher contaminant load typically associated with sludge feedstocks, which accumulate metals during the wastewater treatment process.

To improve the suitability of such a biochar, several strategies could be considered. For raw manure biochar, reducing Ni inputs at the feedstock stage (e.g. through careful manure management and minimising contamination from bedding or feed additives) could shift the product back into a compliant class. For biologically treated sewage sludge digestate biochar, options include selective removal of Zn and Ni during wastewater treatment (Bağdat et al., 2022), Blending with clean biomass during pyrolysis (Wang et al., 2022) to dilute heavy-metal concentrations, or post-pyrolysis treatments such as acid washing (Rangabhashiyam et al., 2021) or magnetic separation to reduce the mobility of these metals. Alternatively, targeted application to non-food crops, reclamation soils, or carbon sequestration could offer safe pathways for utilisation where direct entry into the food chain is restricted.

### Influence of feedstock type on P availability

Our results confirm that P (P) availability in biochar is mainly dependent on the feedstock origin and mineral content, supporting H1. Manure-derived biochar was primarily composed of Ca–P minerals, including hydroxyapatite and whitlockite (Cao & Harris, 2010; Sun et al., 2022), and brushite, which are moderately soluble and significantly contribute to crop uptake, especially in wheat and spinach. Specifically, raw manure biochar was dominated by calcite (CaCO_3_), which, although not a phosphorus-bearing mineral, indirectly enhances phosphorus availability by increasing soil pH, reducing Al and Fe activity, and promoting the dissolution and redistribution of Ca–P phases, thereby facilitating greater phosphorus accessibility to plant roots. On the other hand, sewage sludge biochar contained high levels of Fe and Al-bound P forms (e.g., berlinite, variscite, augelite, and iron phosphate phases), which were mainly insoluble under agricultural conditions and showed a weak relationship with plant uptake. Nevertheless, measurable P uptake was observed, likely supported by minor Ca and Mg-associated P phases and amorphous or poorly ordered phosphorus pools not resolved by XRD, which can be mobilized by extraction methods and rhizosphere processes. This aligns with earlier reports indicating that Ca-bound P phases in manure biochar are more accessible, mainly when produced at moderate pyrolysis temperatures (around 400 °C) (Jin et al., 2016; Musa et al., 2024). In contrast, Fe/Al-bound P in sewage sludge biochar remains as less soluble compounds that are less accessible to crops but can increase the total P content (Figueiredo et al., 2021; Filho et al., 2024; Melia et al., 2019). Mineralogical evidence from XRD (Fig. 1), FTIR (Fig. 2), and SEM (Fig. 3) further showed that sewage sludge biochar has compact structures, which limit P solubilisation. Li et al., (2018) reported similar findings, and Figueiredo et al., 2021) observed that sludge-derived biochar contains more amorphous Fe/Al–P complexes, resulting in a lower fertiliser value despite a high total P content.

### Relationship between biochar-extracted P and crop-specific uptake (wheat, faba bean, and spinach)

The relationship between biochar-applied P(P), as estimated by different extraction methods, and crop uptake varied substantially across crops, extractants, and feedstocks. Importantly, these relationships were strongly influenced by feedstock-specific interactions, and averaging extraction performance across feedstocks can obscure critical differences in agronomic relevance. This was evident from the large slope differences, response ratios (RR), and log response ratios (LRR) observed between sludge-derived biochar and manure-derived biochar across several extractants, highlighting the importance of considering feedstock-specific dependencies when evaluating extraction performance (Appendix A1, Appendix A1, Appendix A1).

In wheat grain, nitric acid digestion provided the strongest relationship with both seed and total P uptake (R^2^ = 0.59–0.66, *P* < 0.001) and showed minimal slope divergence between feedstocks. For example, in total wheat uptake, slopes were similar for biochars derived from biologically treated sewage sludge digestate and digestate manure (RR = 0.88; LRR = − 0.13; 14% difference), indicating consistent predictive performance across biochar origins. However, this is partly driven by the experimental design, as biochar application rates were based on total P measured by nitric acid digestion. Thus, its predictive strength reflects total P input rather than plant-available P.

Intermediate relationships were observed with citric acid, dithionite, and Mehlich-3 extractions (R^2^ = 0.42–0.49). Although these extractants exhibited strong feedstock-dependent slope divergence, as reflected by substantial response ratios and LRR values (RR = 0.48–5.16; LRR = − 0.74 to 1.64), indicating that their predictive strength varied considerably between biologically treated sewage sludge digestate and digestate manure-derived biochars. Olsen, ammonium lactate, and water extractions produced slightly weaker but still significant fits (R^2^ = 0.38–0.45) yet showed extremely large slope differences (RR = 10.00–18.97; LRR = 2.30–2.94), demonstrating strong sensitivity to feedstock-specific differences in P lability. In contrast, oxalate showed the poorest overall predictive performance (R^2^ = 0.27–0.30), despite exhibiting large slope divergence (RR = 0.38; LRR = − 0.97), indicating limited capacity to reliably explain wheat uptake across feedstocks. These results suggest that while nitric acid captures more P recovery potential, agronomic extractants like Mehlich-3 and ammonium lactate more closely approximate the labile pools accessible to cereals (Basak, 2019; Kiani & Ylivainio, 2024; Morais et al., 2023; Ylivainio et al., 2021).

For faba bean, ammonium lactate, Mehlich-3, nitric acid digestion, Olsen, and water extractions showed significant correlations with total uptake (R^2^ = 0.41–0.53). In contrast, seed uptake was best explained by nitric acid digestion (R^2^ = 0.54, *P* < 0.001), suggesting that total P content played a dominant role in determining P allocation to reproductive tissues.However, slope comparisons revealed extremely strong feedstock-dependent interactions across all extractants, with response ratios ranging from 0.15 to 12.73 and LRR values ranging from − 1.92 to 2.54. Notably, Olsen extraction showed exceptionally high responsiveness in sludge-derived biochar relative to manure-derived biochar (RR = 12.73; LRR = 2.54), indicating strong sensitivity to feedstock-specific Ca–P and Fe–P mineral forms. In contrast, citric acid, dithionite, and oxalate consistently showed poor or non-significant relationships (R^2^ ≤ 0.04) despite large response ratios, suggesting that these extractants mobilize P pools that are not consistently accessible to faba bean. These results reflect faba bean’s reliance on moderate labile pools, which can be mobilised through rhizosphere acidification and organic acid exudation (Tesfaye et al., 2021; Zwieten et al., 2015). The relatively strong performance of ammonium lactate and Mehlich-3 suggests their suitability as practical predictors of P availability in legume systems. Biochar feedstock differences further contributed to the divergent P uptake patterns observed between crops. Sewage sludge biochar provided a similar pool of Fe/Al-bound P for both crops, mainly in the form of poorly soluble complexes such as variscite and strengite, which limited their agronomic effectiveness. In contrast, the manure biochar differed by crop. In the faba bean system, the biochar was derived from raw manure, which retained more crystalline Ca–P minerals such as hydroxyapatite and whitlockite. These phases are sparingly soluble but can be mobilised by faba bean through rhizosphere acidification and organic acid exudation, leading to a stronger link between uptake and extractants targeting moderately labile pools (e.g., AL, Mehlich-3). By contrast, the wheat experiment used biochar from digested manure, where anaerobic digestion reduced Ca–P crystallinity and introduced more amorphous and intermediate P phases, as shown by the results from the XRD analysis. Since wheat lacks the same capacity to acidify its rhizosphere or release large amounts of organic acids (Kidd et al., 2023), its uptake was less influenced by moderate labile pools and more strongly related to the total P content. This explains why the predictive strength of extraction methods differed between the two crops, reflecting both the feedstock mineralogy and the plant’s nutrient acquisitimanuscripton strategies.

The trends were reversed in spinach, a leafy crop with a shallow root system and high demand for immediately soluble P, agronomic extractants including ammonium lactate, Mehlich-3, Olsen, and water extractions showed the strongest correlations with uptake (R^2^ = 0.75–0.76, *P* < 0.001), along with extremely large response ratios (RR = 3.14–17.73; LRR = 1.14–2.88). These results indicate that spinach uptake is primarily governed by soluble and weakly bound P fractions. In contrast, nitric acid digestion failed to explain crop response (R^2^ < 0.01, P = 0.729). This suggests that spinach uptake is mainly dependent on soluble and weakly bound P (P) pools, which are best assessed by agronomic soil tests. In contrast, strong acid digestion mobilizes phases not accessible to spinach in the short term (Sepúlveda-Cadavid et al., 2021). Citric acid, dithionite, and oxalate again showed weak or inconsistent fits (R^2^ = 0.17–0.28), reflecting their limited relevance to spinach nutrition.

Taken together, these findings demonstrate that extraction performance cannot be reliably inferred from averaged relationships alone, as strong feedstock-dependent interactions significantly influence predictive accuracy. Taken together, these findings demonstrate that extraction performance cannot be reliably inferred from averaged relationships alone, as strong feedstock-dependent interactions significantly influence predictive accuracy. Nitric acid digestion, Mehlich-3, and ammonium lactate best explained wheat and faba bean uptake, while Mehlich-3, ammonium lactate, Olsen, and water extractions were most relevant for spinach. This highlights the need for crop and feedstock-specific evaluation of extraction methods. For agronomic purposes, Mehl ich 3 and AL appear to be reasonable extractants across crop types, suggesting that the standard AL method used in Norway is a suitable approach for these systems. At the same time, nitric acid is valuable for estimating total recovery potential. These insights underscore the importance of aligning P testing methods with both fertiliser characteristics and crop requirements to enhance the prediction of biochar fertiliser performance across various production systems.

### Towards standardized biochar P testing

Taken together, our findings suggest that biochar P testing can be operationalised through a stepwise, tiered approach. First, Tier 1 analysis using nitric acid digestion should be applied to all biochars to quantify total P recovery and to establish application rates for nutrient budgeting and regulatory purposes. Second, the feedstock origin should be identified, distinguishing between manure-derived biochars, which are typically dominated by Ca-associated P forms, and sewage sludge biochars, where P is primarily bound to Fe and Al phases with low short-term bioavailability. In Tier 2, agronomic extractants should then be selected accordingly. For manure biochars, ammonium lactate (P-AL), the standard method used in Norwegian fertilizer planning, and Mehlich-3 are appropriate for estimating crop-relevant, moderately labile P pools. Water or Olsen extraction is additionally recommended for leafy crops with high demand for immediately soluble P. In contrast, for sewage sludge digestate biochars, P-AL and Mehlich-3 should be used primarily as screening tools, recognising their limited ability to predict crop P uptake due to the dominance of chemically stable Fe/Al-bound P. Finally, Tier-2 results should always be interpreted in a crop-specific context, acknowledging that low extractable P in sludge-derived biochars reflects intrinsic chemical constraints of the material rather than shortcomings of the extraction method itself.

## Conclusions

It was possible to conclude that manure-derived biochar had higher agronomic value than sewage sludge biochar, which was limited by Fe/Al-bound P and heavy-metal contamination. Nitric acid digestion best predicted wheat and faba bean P uptake, whereas agronomic extractants (Mehlich-3, ammonium lactate, Olsen, water) correlated most strongly with spinach uptake. However, in general, for agronomic purposes, Mehlich-3 and AL appear to be reasonable extractants across crop types, providing reliable estimates of plant-available P while also capturing feedstock-specific differences in P lability. Digestate manure biochar met regulatory requirements, but raw manure and sewage sludge biochar exceeded Ni and Zn thresholds, respectively. These findings emphasise the need for a tiered approach to biochar P testing and for regulatory frameworks that balance environmental safety with the potential for nutrient recycling.

## Future work and take-home message

***Future work*** Future studies should (I) test additional P extraction methods beyond those compared here; (II) include other soil types to assess soil-specific interactions with biochar; (III) explore blending strategies (e.g., manure + clean biomass) to dilute contaminants in sludge-derived biochar; and (IV) investigate Ni and Zn removal or immobilization strategies during or after pyrolysis.

***Take-home message*** Digestate manure biochar is the most promising option, providing balanced nutrients, predictable P availability, and regulatory compliance. Sludge-derived biochar is limited by Ni and Zn burdens but blending and removal strategies could improve their suitability. A tiered P testing approach (acid digestion + agronomic extractants) provides the most reliable framework for predicting crop responses and informing regulations. Aligning feedstock management, testing protocols, and policy will be essential to realize biochars safe and effective role in sustainable P management.

## Supplementary Information

Below is the link to the electronic supplementary material.

Supplementary file1 (DOCX 26 kb)

## Data Availability

“The datasets generated during and/or analysed during the current study are not publicly available due to [DATA ARE STORED INTERNALLY AT NMBU DATA DRIVE AND SHARED AMONG THE PARTNERS, NOT PUBLIC] but are available from the corresponding author on reasonable request.”
