# Supplementary material for: Evaluating plant-available P methods and quality characterization of biochar derived from manure and sewage sludge
Source: Environ Geochem Health. 2026 Mar 27;48(6):266. doi: 10.1007/s10653-026-03157-0 (PMC13031242; doi:10.1007/s10653-026-03157-0)
Supplement: Supplementary file 1 — Supplementary file1 (DOCX 26 kb) [file 10653_2026_3157_MOESM1_ESM.docx]

**Appendix A**

**Appendix A1** Relationship between extractable phosphorus and total wheat P uptake: Comparison of BBSWD and BDM using slopes, response ratios (RR), and log response ratios (LRR)

| **Extraction Method** | **BBSWD**  **Slope** | **BDM**  **Slope** | **Response Ratio** | **LRR** | **% Difference** | **Interaction Strength** | **Average R²** | **Average**  **value** |
| --- | --- | --- | --- | --- | --- | --- | --- | --- |
| Ammonium lactate | 0.19 | 0.019 | 10.00 | 2.30 | 900% | Extremely strong | 0.45 | 0.002 |
| Nitric acid digestion | 0.014 | 0.016 | 0.88 | -0.13 | 14% | Very weak | 0.66 | <0.001 |
| Olsen | 3.8 | 0.36 | 10.56 | 2.36 | 956% | Extremely strong | 0.44 | 0.003 |
| Dithionite | 0.0067 | 0.014 | 0.48 | -0.74 | 109% | Very strong | 0.46 | 0.002 |
| Citric acid | 0.021 | 0.040 | 0.53 | -0.64 | 90% | Strong | 0.49 | 0.001 |
| Mehlich-3 | 0.32 | 0.062 | 5.16 | 1.64 | 416% | Extremely strong | 0.47 | 0.002 |
| Oxalate | 0.0057 | 0.015 | 0.38 | -0.97 | 163% | Very strong | 0.30 | 0.019 |
| Water | 11 | 0.58 | 18.97 | 2.94 | 1797% | Extremely strong | 0.43 | 0.003 |

***Where*** *BBTSSD, Biochar from biologically treated sewage sludge-digestate;BDM, Biochar from digestate manure*

**Table A2.** Relationship between extractable phosphorus and total faba bean P uptake: Comparison of BBSWD and BRM using slopes, response ratios (RR), and log response ratios (LRR)

| **Extraction Method** | **BBSWD**  **Slope** | **BEM Slope** | **Response Ratio** | **LRR** | **% Difference** | **Interaction Strength** | **Average R²** | **Average**  **value** |
| --- | --- | --- | --- | --- | --- | --- | --- | --- |
| Nitric acid digestion | 0.05 | 0.097 | 0.52 | -0.66 | 94% | Strong | 0.45 | 0.002 |
| Mehlich-3 | 0.12 | 0.43 | 0.28 | -1.28 | 258% | Extremely strong | 0.53 | <0.001 |
| Ammonium lactate | 0.72 | 0.23 | 3.13 | 1.14 | 213% | Extremely strong | 0.51 | <0.001 |
| Olsen | 14 | 1.1 | 12.73 | 2.54 | 1173% | Extremely strong | 0.41 | 0.004 |
| Citric acid | 0.075 | 0.30 | 0.25 | -1.39 | 300% | Extremely strong | 0.04 | 0.403 |
| Dithionite | 0.025 | 0.17 | 0.15 | -1.92 | 580% | Extremely strong | <0.01 | 0.814 |
| Oxalate | 0.091 | 0.021 | 4.33 | 1.47 | 333% | Extremely strong | 0.03 | 0.478 |
| 42 | 2.6 | 16.15 | 2.78 | 1515% | Extremely strong | 0.41 | 0.004 | 42 |

***Where*** *BBTSSD, Biochar from biologically treated sewage sludge-digestate; BRM, biochar from raw manure.*

**Table A3.** Relationship between extractable phosphorus and spinach P uptake: Comparison of BBSWD and BRM using slopes, response ratios (RR), and log response ratios (LRR)

| **Extraction Method** | **BBSWD Slope** | **BEM**  **Slope** | **Response Ratio** | **LRR** | **% Difference** | **Interaction Strength** | **Average R²** | **Average**  **value** |
| --- | --- | --- | --- | --- | --- | --- | --- | --- |
| Nitric acid digestion | 0.0046 | 0.0079 | 0.58 | -0.54 | 72% | Strong | <0.01 | 0.729 |
| Ammonium lactate | 0.066 | 0.019 | 3.47 | 1.25 | 247% | Extremely strong | 0.76 | <0.001 |
| Citric acid | 0.025 | 0.0071 | 3.52 | 1.26 | 252% | Extremely strong | 0.017 | 0.092 |
| Dithionite | 0.0023 | 0.014 | 0.16 | -1.81 | 509% | Extremely strong | 0.28 | 0.024 |
| Mehlich-3 | 0.11 | 0.035 | 3.14 | 1.14 | 214% | Extremely strong | 0.75 | <0.001 |
| Olsen | 1.3 | 0.091 | 14.29 | 2.66 | 1328% | Extremely strong | 0.75 | <0.001 |
| Oxalate | 0.019 | 0.0075 | 2.53 | 0.93 | 153% | Very strong | 0.19 | 0.071 |
| Water | 3.9 | 0.22 | 17.73 | 2.88 | 1673% | Extremely strong | 0.75 | <0.001 |

***Where*** *BBTSSD, Biochar from biologically treated sewage sludge-digestate; BRM, biochar from raw manure.*
